# Double Rotational Flaps for Scalp Reconstruction: A Three-Case Series

**DOI:** 10.7759/cureus.116325

**Published:** 2026-09-15

**Authors:** Ludmila Rodrigues Ribeiro da Silva

**Affiliations:** 1 Burn Reconstructive Surgery, Shriners Hospitals for Children, Galveston, USA

**Keywords:** basal cell carcinoma of scalp, double rotational flap, flap necrosis, local scalp flap, scalp cancer, scalp hair, scalp lesions, scalp neoplasm, scalp reconstruction, scalp tumor

## Abstract

Scalp reconstruction after oncologic resection remains challenging because of limited scalp elasticity and the need for adequate vascular perfusion. Aging and androgenetic alopecia are associated with well-described biological and structural changes in the scalp; however, whether these changes have clinically meaningful effects on local flap perfusion, viability, or postoperative healing remains uncertain. This series of three cases describes patients undergoing scalp tumor excision followed by reconstruction using double rotational flaps in similar anatomical regions. Case 1 involved a 56-year-old man with a hair-bearing scalp and nodular basal cell carcinoma. The surgical specimen had a documented scalp thickness of 0.9 cm. Postoperative healing occurred without complications. Case 2 involved an 88-year-old bald man with advanced androgenetic alopecia and invasive squamous cell carcinoma. The surgical specimen measured 2.9×2.8 cm and had a documented scalp thickness of 0.4 cm. Closure was achieved under clinically assessed moderate tension. The patient developed an approximately 1×5 cm area of superficial distal flap necrosis, which was managed conservatively with collagenase ointment and local wound care, with secondary healing by postoperative day 54 without reoperation. Case 3 involved a 73-year-old woman with a hair-bearing scalp and invasive moderately differentiated squamous cell carcinoma. The surgical specimen measured 2.9×2.5×0.5 cm, with tumor invasion extending to the deep dermis and evidence of perineural invasion. The documented scalp thickness was 0.5 cm. Postoperative healing occurred without flap necrosis or wound dehiscence.

These observations raise the hypothesis that factors beyond measured scalp thickness may warrant further investigation in scalp reconstruction. However, the different postoperative outcomes cannot be attributed to androgenetic alopecia or scalp tissue characteristics because vascular perfusion and tissue quality were not objectively measured, and patient- and procedure-related factors, including closure tension, may have contributed. Prospective studies incorporating objective assessments of scalp tissue characteristics, perfusion, biomechanics, and relevant clinical and technical variables are needed to evaluate this hypothesis.

## Introduction

Scalp reconstruction remains challenging because of limited scalp elasticity, firm adherence of the galea aponeurotica, the convex calvarial surface, and restricted tissue mobility compared with other anatomical regions [1-6]. Successful reconstruction requires durable soft tissue coverage while preserving scalp contour, vascularity, hair-bearing tissue, and aesthetic appearance. Selection of reconstructive technique depends on defect characteristics, scalp anatomy, patient comorbidities, tissue availability, and reconstructive goals [1-7].

Cutaneous malignancies, particularly basal cell carcinoma (BCC) and squamous cell carcinoma (SCC), are common indications for scalp reconstruction in elderly patients [2,4-7]. Following oncologic resection, reconstructive planning is influenced by defect size and location, involvement of the periosteum or calvarium, previous surgery or radiotherapy, scalp laxity, and characteristics of the surrounding tissue [1-8].

Reconstructive options include primary closure, healing by secondary intention, skin grafting, local advancement or rotational flaps, tissue expansion, regional flaps, and free tissue transfer [1-8]. When adequate adjacent tissue is available, local flaps offer favorable color and texture match, preservation of hair-bearing scalp, and avoidance of the donor-site morbidity associated with more complex reconstructive options [1-8]. A double rotational flap uses two opposing local scalp flaps rotated toward a central defect to redistribute closure tension and recruit adjacent tissue for coverage.

Aging is associated with dermal thinning, extracellular matrix remodeling, reduced elasticity, and changes in cutaneous vascular density that may impair wound healing [9-13]. Androgenetic alopecia is likewise associated with well-described biological changes, including follicular miniaturization and perifollicular remodeling [14-16]. However, whether these changes result in clinically meaningful alterations in local scalp flap perfusion, viability, or postoperative healing has not been established. Accordingly, androgenetic alopecia should not be considered an established independent risk factor for flap necrosis, and its potential relevance to reconstructive outcomes remains hypothetical.

Published clinical series of local scalp flaps have reported favorable reconstructive outcomes when appropriate flap design, preservation of vascular supply, adequate tissue mobilization, and minimization of closure tension are achieved [17-20]. These studies have primarily focused on defect characteristics, flap geometry, vascular anatomy, and surgical technique rather than on whether biological or structural characteristics of the recipient scalp are associated with reconstructive outcomes [17-20]. Although baldness is recognized as a risk factor for scalp skin cancer in the setting of cumulative ultraviolet exposure, whether scalp characteristics associated with androgenetic alopecia have clinically meaningful implications for local flap healing remains unknown [15].

This case series describes three patients with scalp malignancies who underwent tumor excision followed by reconstruction using double rotational flaps. The cases provide a hypothesis-generating comparison of postoperative healing among patients with different scalp characteristics and measured scalp thicknesses. Rather than establishing androgenetic alopecia or tissue characteristics as independent determinants of flap survival, this series raises the hypothesis that scalp characteristics beyond measured thickness may warrant further investigation in reconstructive planning.

## Case presentation

All three defects were reconstructed using the same general double rotational flap technique. The rotational flaps measured approximately 3.5 cm in width and 6 cm in length. Undermining was limited to the planned rotational flaps and was not extended into the surrounding adjacent scalp. Galeal scoring was performed in all three cases to increase flap mobility and facilitate closure. Closure tension was assessed clinically rather than objectively. None of the three patients were smokers. None of the patients were receiving chronic corticosteroids, immunosuppressive therapy, systemic anticancer therapy, or other medications known to significantly impair wound healing.

Case 1 - younger hair-bearing scalp

A 56-year-old man presented with nodular basal cell carcinoma in a hair-bearing scalp region. We performed tumor excision followed by immediate reconstruction using double rotational flaps. According to the official pathology report, histopathological examination confirmed nodular basal cell carcinoma. Macroscopic examination of the surgical specimen documented an irregular skin fragment measuring 3.0×2.7 cm with a scalp thickness of 0.9 cm. The lesion measured 2.0×1.7 cm and was located 0.5 cm from the surgical margin. Postoperative healing occurred uneventfully without necrosis, infection, or wound dehiscence (Figures 1-3).

**Figure 1:**
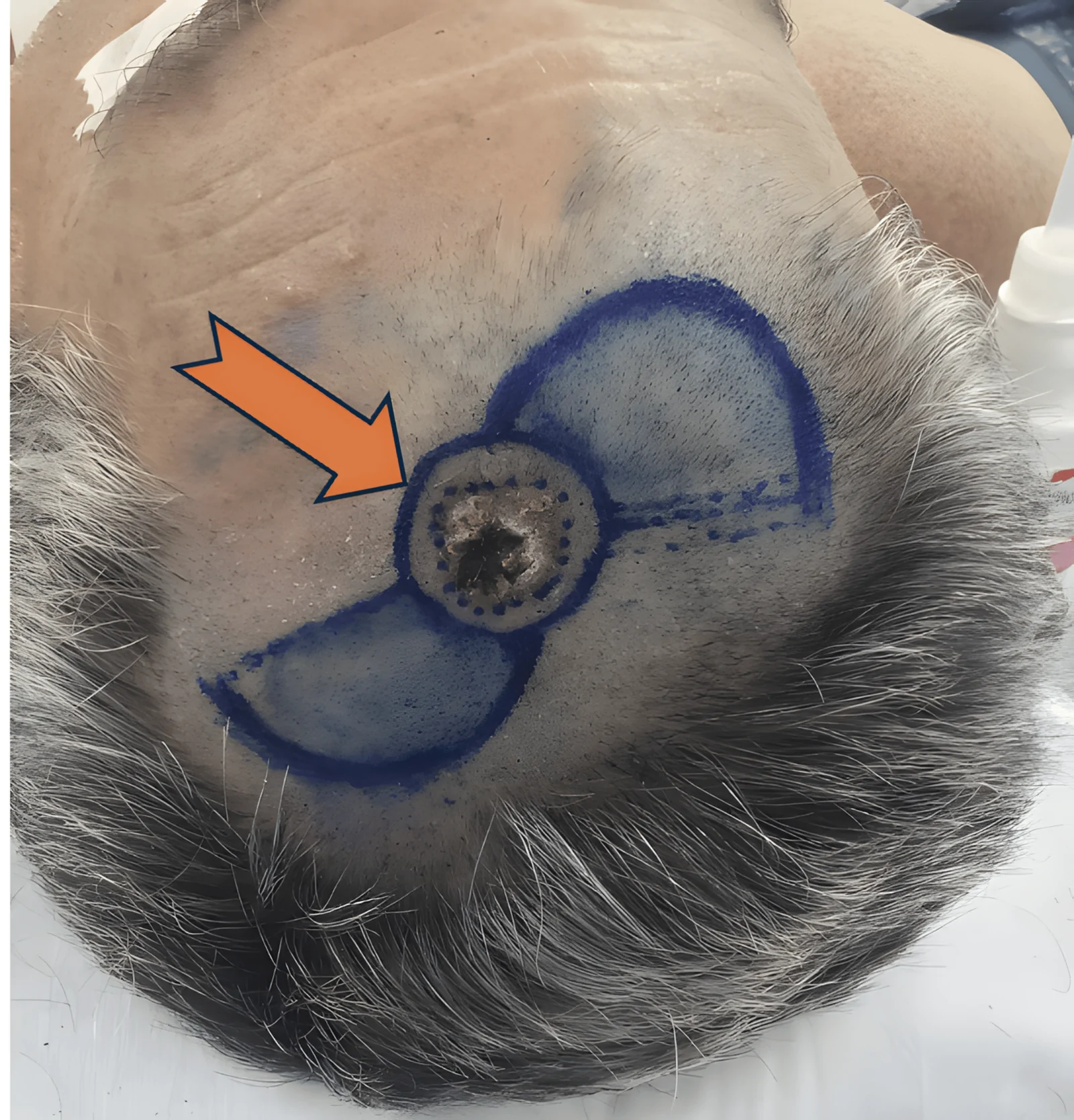
Preoperative marking (arrow) of a scalp skin cancer in a 56-year-old man with hair-bearing scalp, planned for excision and reconstruction using a double rotational flap. The photograph demonstrates the hair-bearing characteristics of the surrounding scalp before reconstruction.

**Figure 2:**
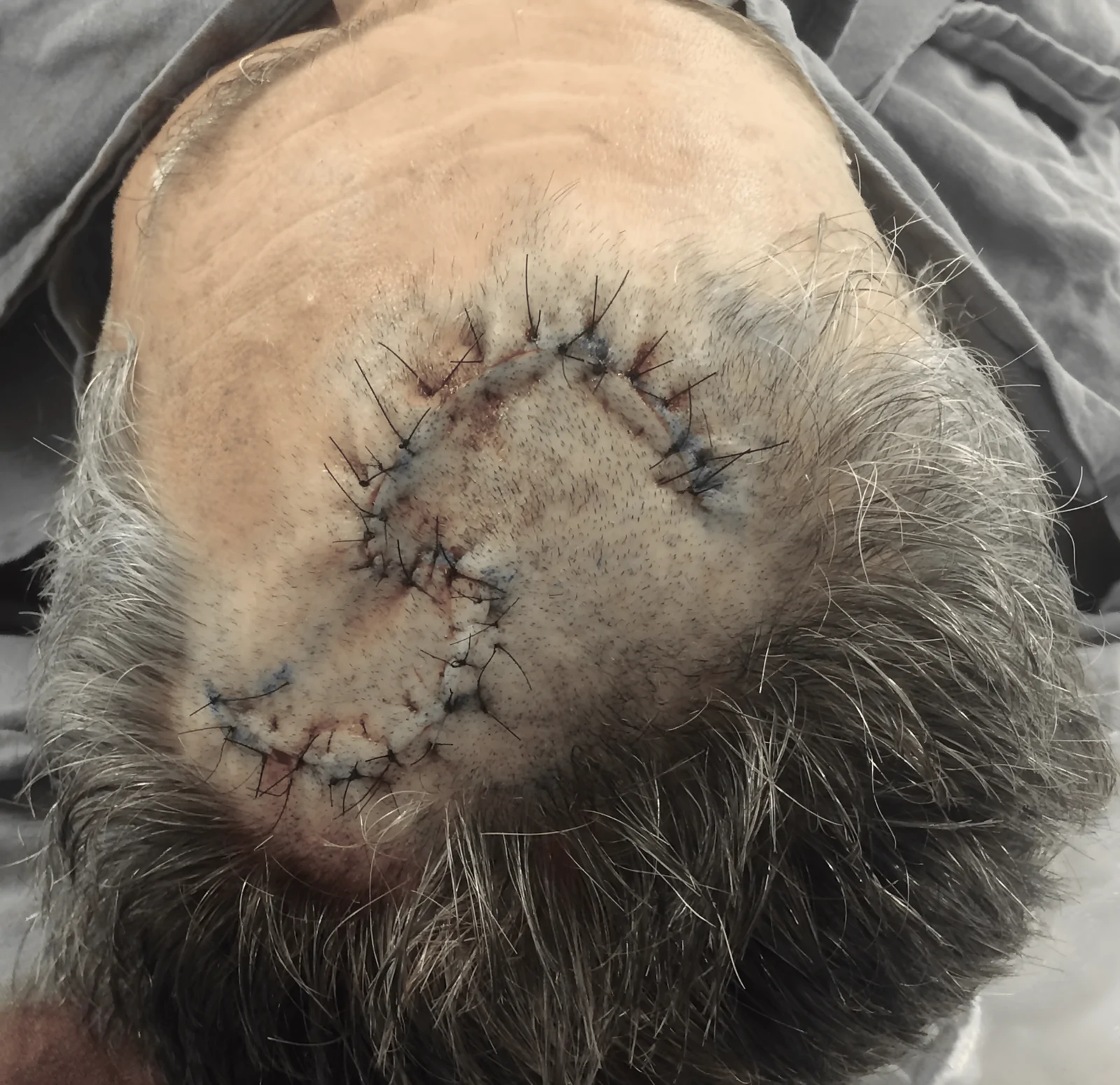
Immediate postoperative result following double rotational flap reconstruction in a 56-year-old patient with hair-bearing scalp.

**Figure 3:**
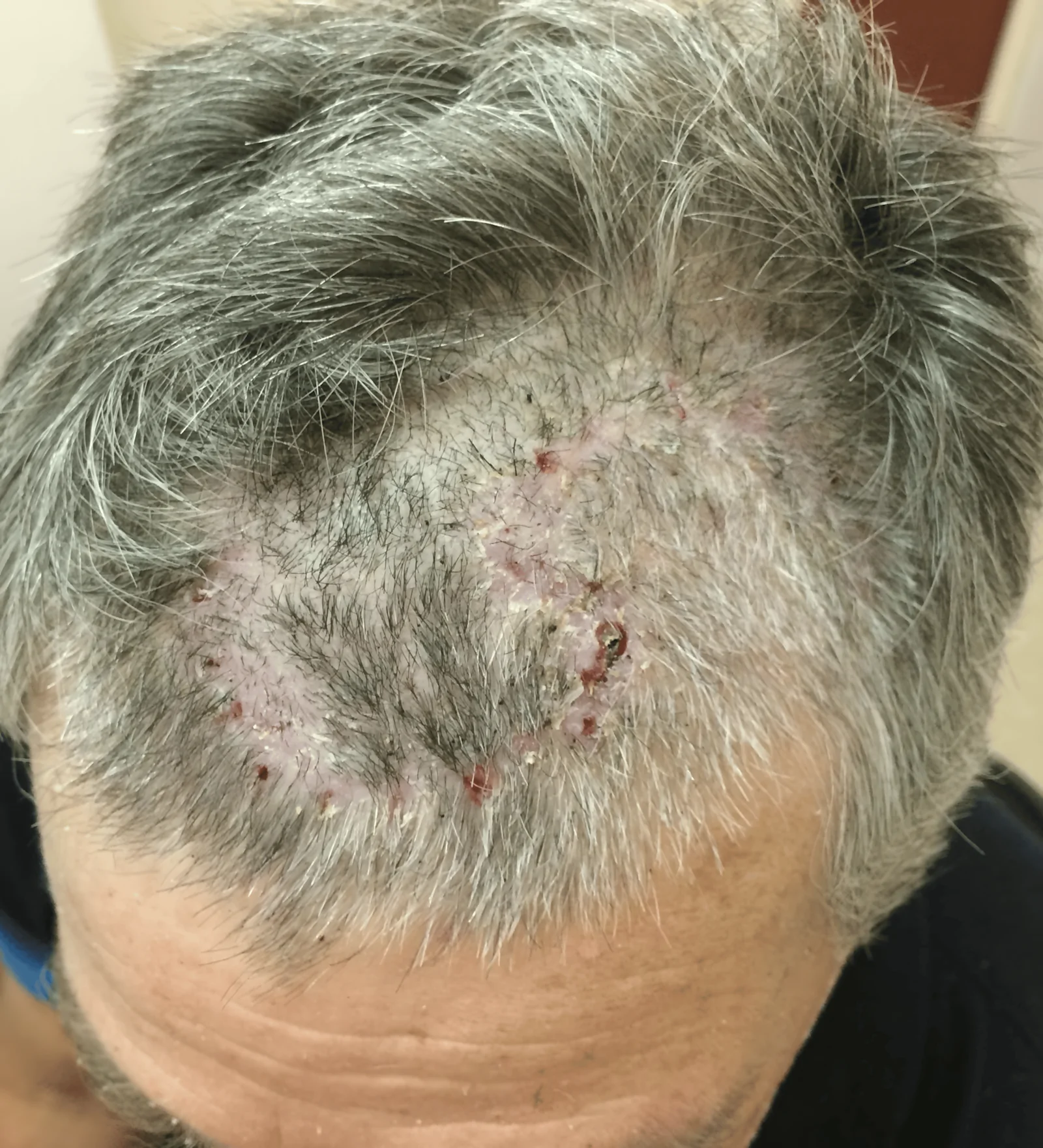
Postoperative day 14 following excision of scalp basal cell carcinoma and reconstruction with a double rotational flap in a 56-year-old man with a hair-bearing scalp. The flap demonstrated satisfactory clinical viability and wound healing without evidence of dehiscence, infection, or necrosis. Hair-bearing scalp coverage and contour were maintained.

Case 2 - elderly bald scalp

An 88-year-old bald man with advanced androgenetic alopecia presented with invasive squamous cell carcinoma of the scalp. Following oncologic excision, reconstruction was performed using the double rotational flap technique described above in a similar anatomical region. Closure was achieved under clinically assessed moderate tension. According to the official pathology report, histopathological examination confirmed invasive squamous cell carcinoma. Macroscopic examination of the surgical specimen documented an ovoid skin fragment measuring 2.9×2.8 cm, with a scalp thickness of 0.4 cm. The lesion measured 2.1×1.9 cm and was located 0.4 cm from the surgical margin. Postoperatively, the patient developed an approximately 1×5 cm area of superficial distal flap necrosis, which was managed conservatively with collagenase ointment and local wound care. Progressive secondary healing occurred over approximately 54 days without the need for reoperation (Figures 4-8).

**Figure 4:**
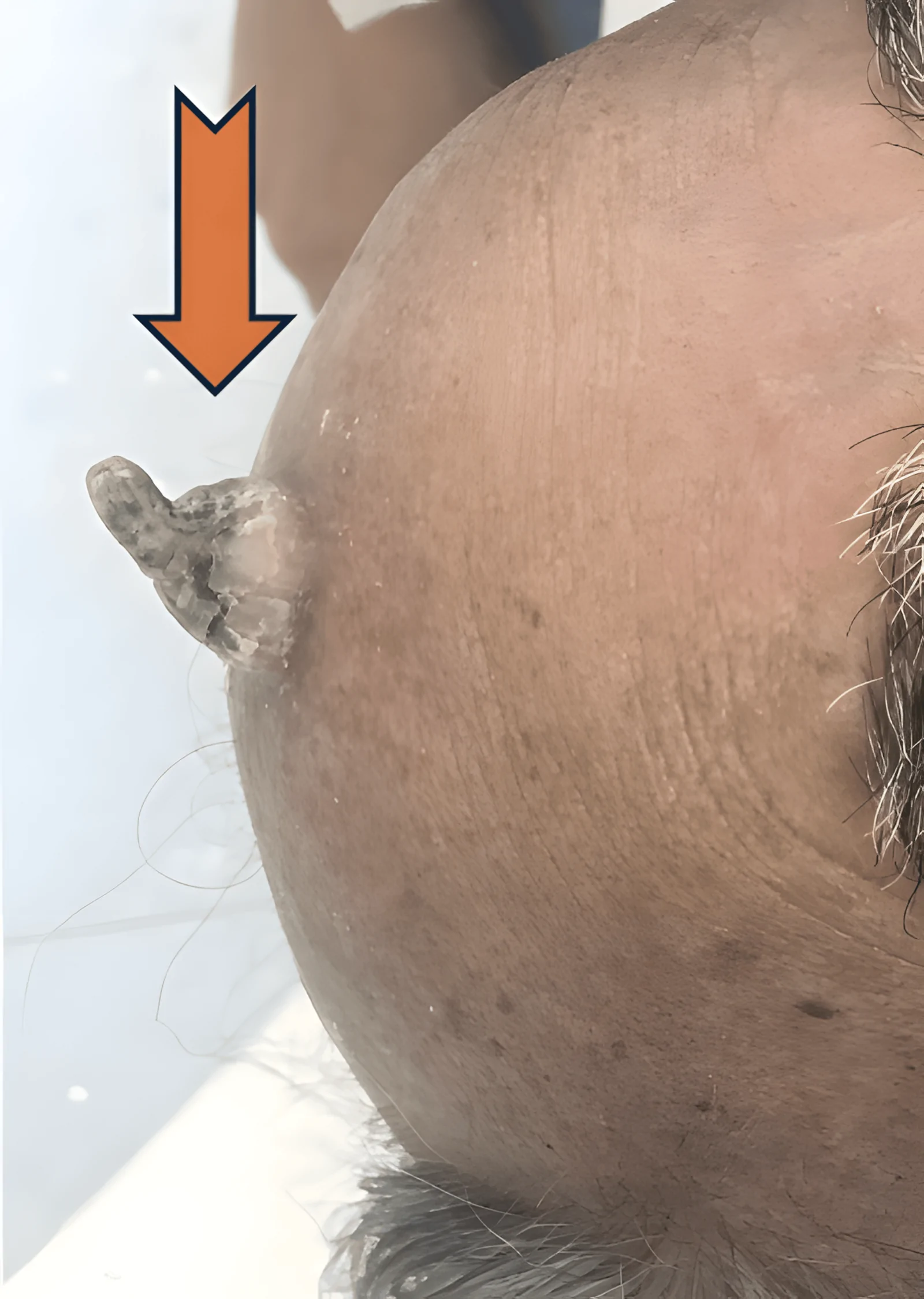
Preoperative clinical photograph of an exophytic scalp squamous cell carcinoma in the frontoparietal region (arrow) of an 88-year-old bald man. The lesion was subsequently excised with oncologic margins and reconstructed using a double rotational scalp flap. The official pathology report documented the diagnosis of squamous cell carcinoma.

**Figure 5:**
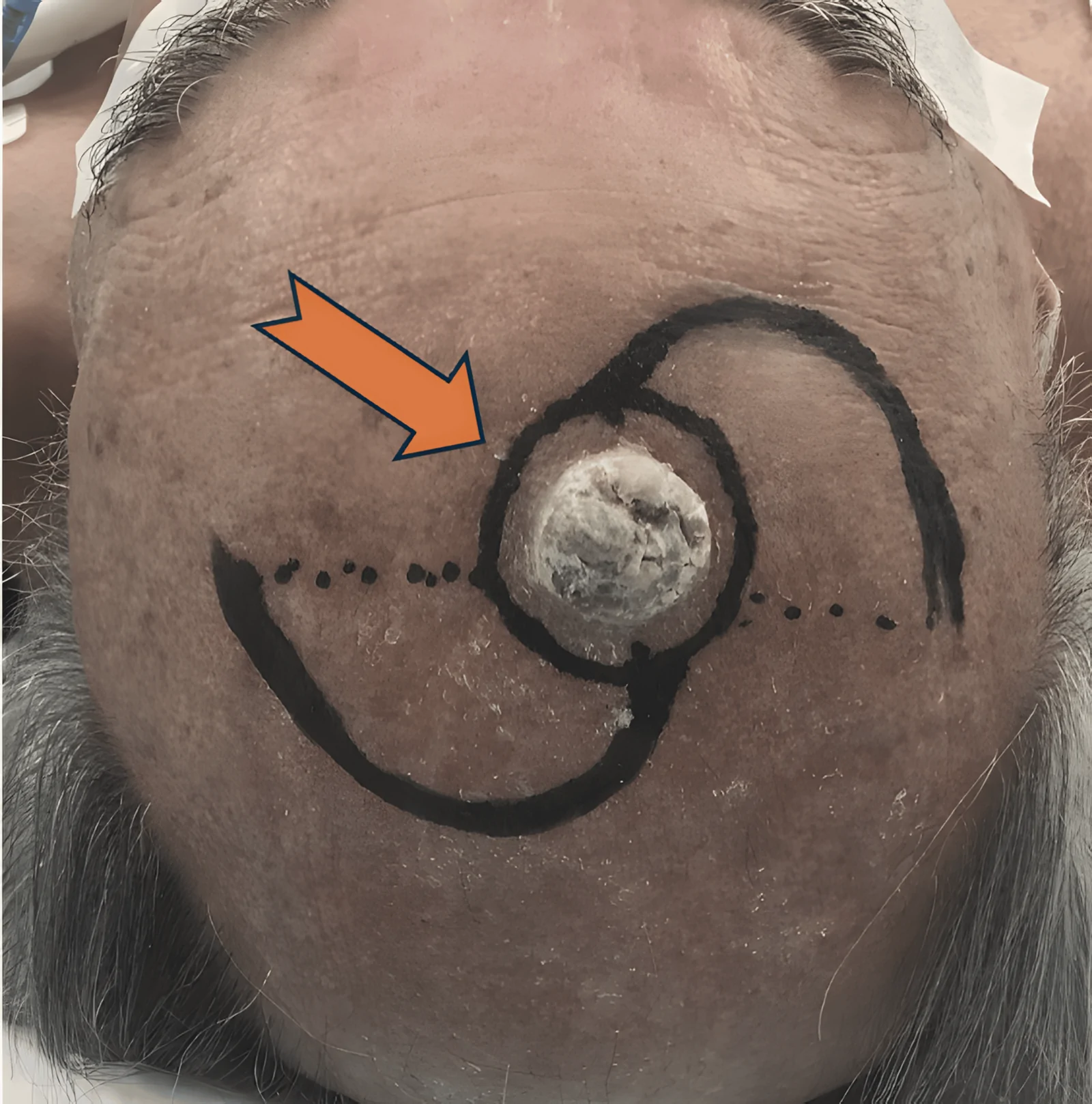
Preoperative marking (arrow) demonstrating the double rotational flap design for scalp reconstruction following excision of squamous cell carcinoma in an 88-year-old bald man. Curvilinear flap markings demonstrate the planned tissue recruitment around the anticipated central defect.

**Figure 6:**
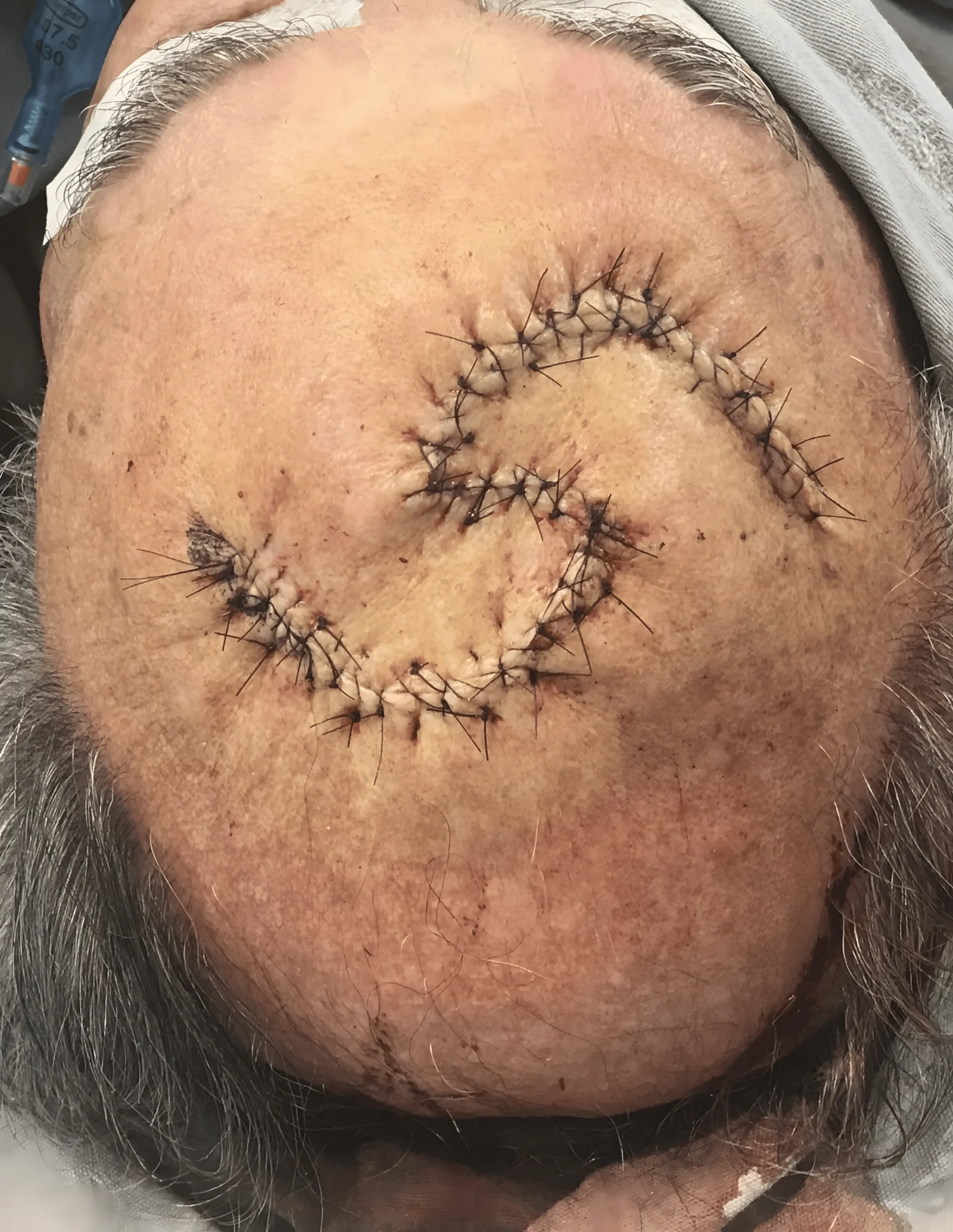
Immediate postoperative appearance following scalp reconstruction with double rotational flaps after excision of squamous cell carcinoma in an 88-year-old bald man. Complete closure of the defect was achieved under clinically assessed moderate tension.

**Figure 7:**
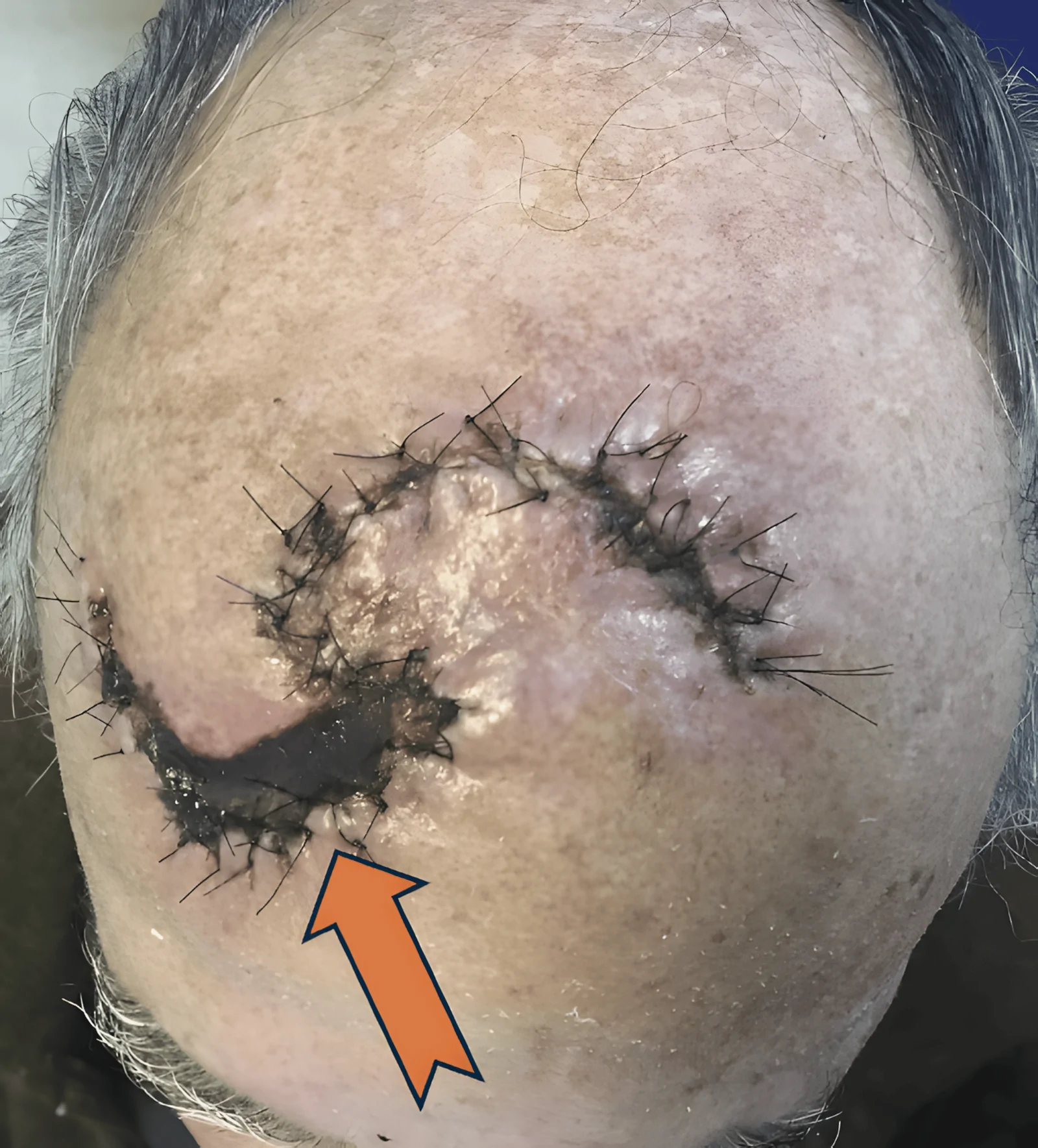
Postoperative day seven following scalp reconstruction with a double rotational flap after excision of squamous cell carcinoma in an 88-year-old bald man. The image demonstrates early superficial ischemic changes and superficial distal flap necrosis (arrow). Conservative management with local wound care and collagenase ointment was initiated.

**Figure 8:**
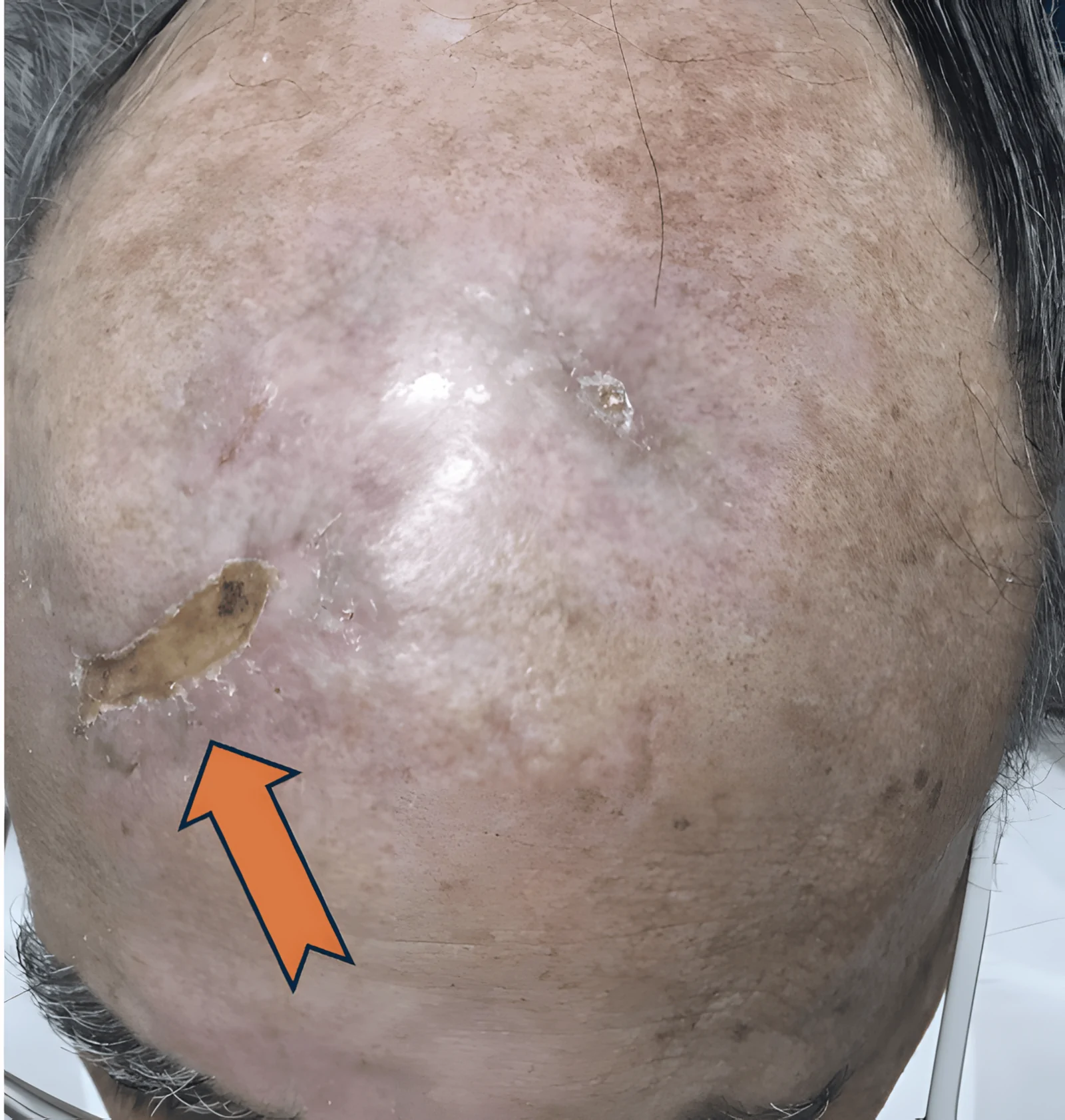
Postoperative day 54 following scalp reconstruction with double rotational flaps after excision of squamous cell carcinoma in an 88-year-old bald man. The patient developed superficial distal flap necrosis (arrow), which was managed conservatively with collagenase ointment and local wound care, resulting in satisfactory secondary healing without the need for reoperation.

Case 3 - elderly female hair-bearing scalp

A 73-year-old woman with a preserved hair-bearing scalp presented with invasive moderately differentiated squamous cell carcinoma of the scalp. According to the official pathology report, histopathological examination confirmed invasive moderately differentiated squamous cell carcinoma. The report documented a discoid skin fragment measuring 2.9×2.5×0.5 cm, with the lesion measuring approximately 2.5 cm in greatest dimension and tumor invasion extending to the deep dermis with evidence of perineural invasion. Angiolymphatic invasion was not identified, and surgical margins were free of neoplasia. Reconstruction was performed using double rotational flaps in a similar scalp region. Postoperative healing occurred without flap necrosis, infection, or wound dehiscence (Figures 9-11). Table 1 summarizes the clinical, pathological, and postoperative characteristics of the three patients.

**Figure 9:**
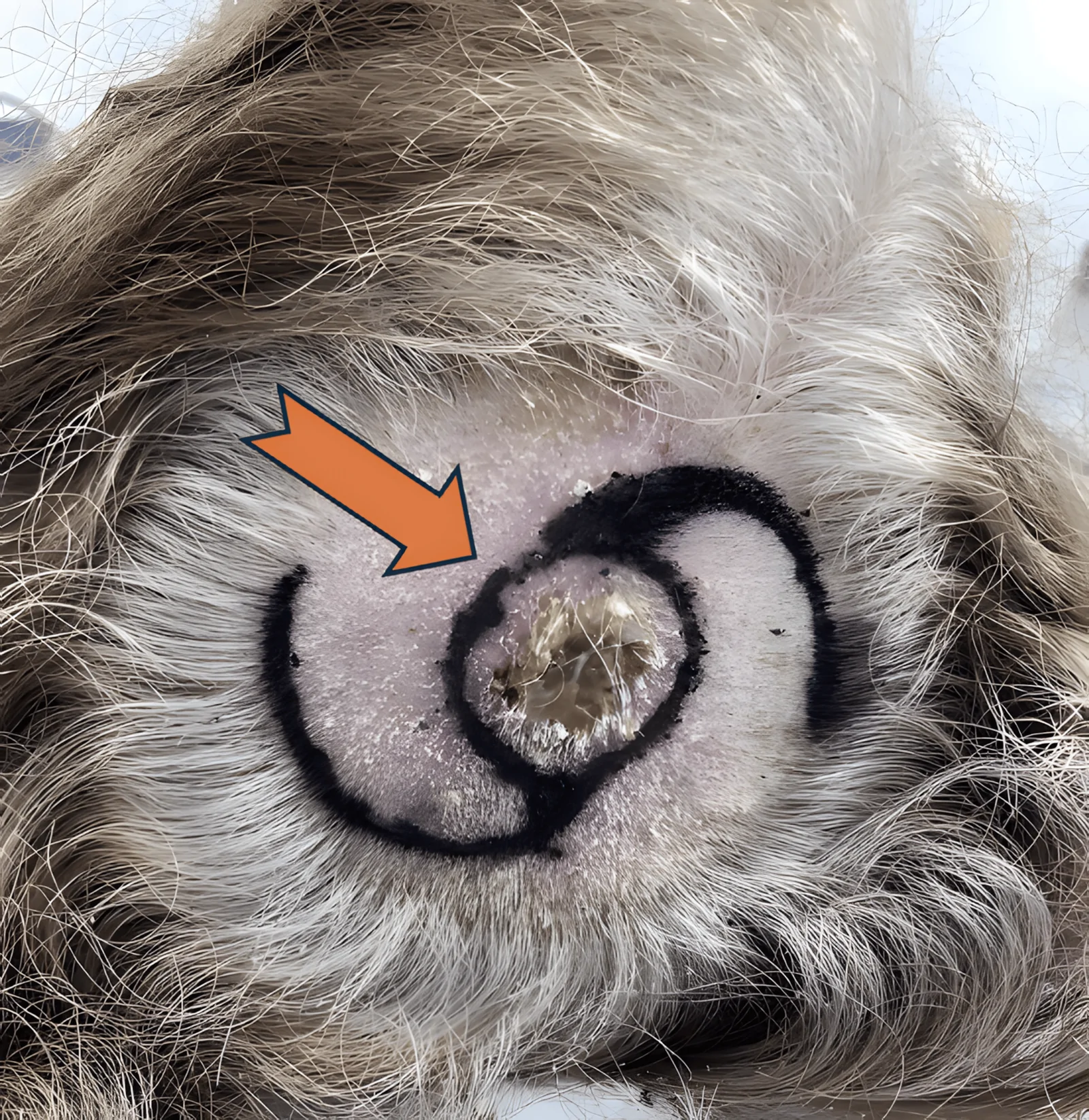
Preoperative surgical markings demonstrating the planned double rotational flap design for scalp defect reconstruction following oncologic excision in a 73-year-old woman. Curvilinear rotational flaps were outlined to facilitate tissue mobilization and tension redistribution around the anticipated central defect (arrow).

**Figure 10:**
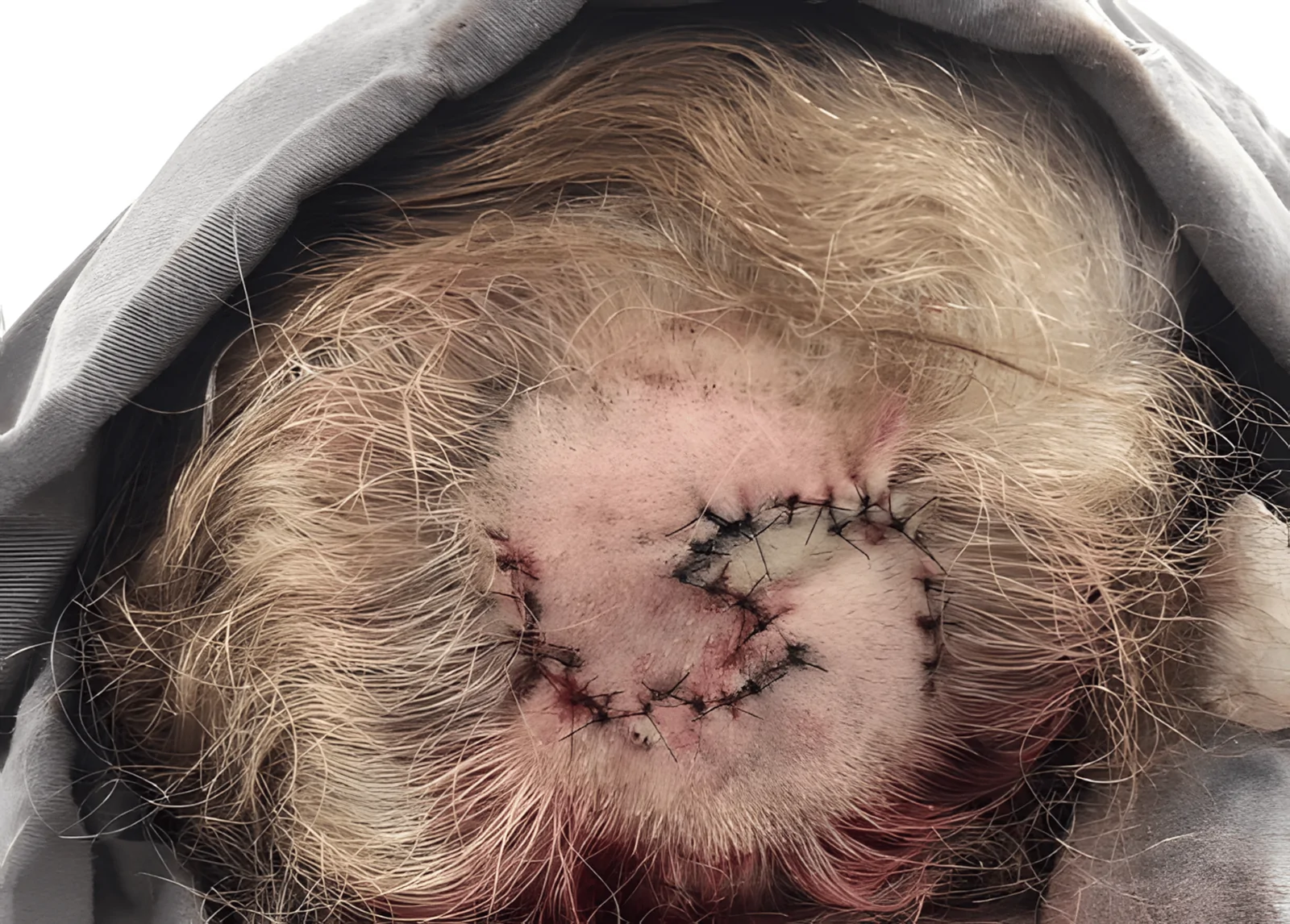
Immediate postoperative appearance demonstrating closure of the scalp defect with a double rotational flap after oncologic excision in a 73-year-old woman, with preservation of the surrounding hair-bearing scalp (arrow).

**Figure 11:**
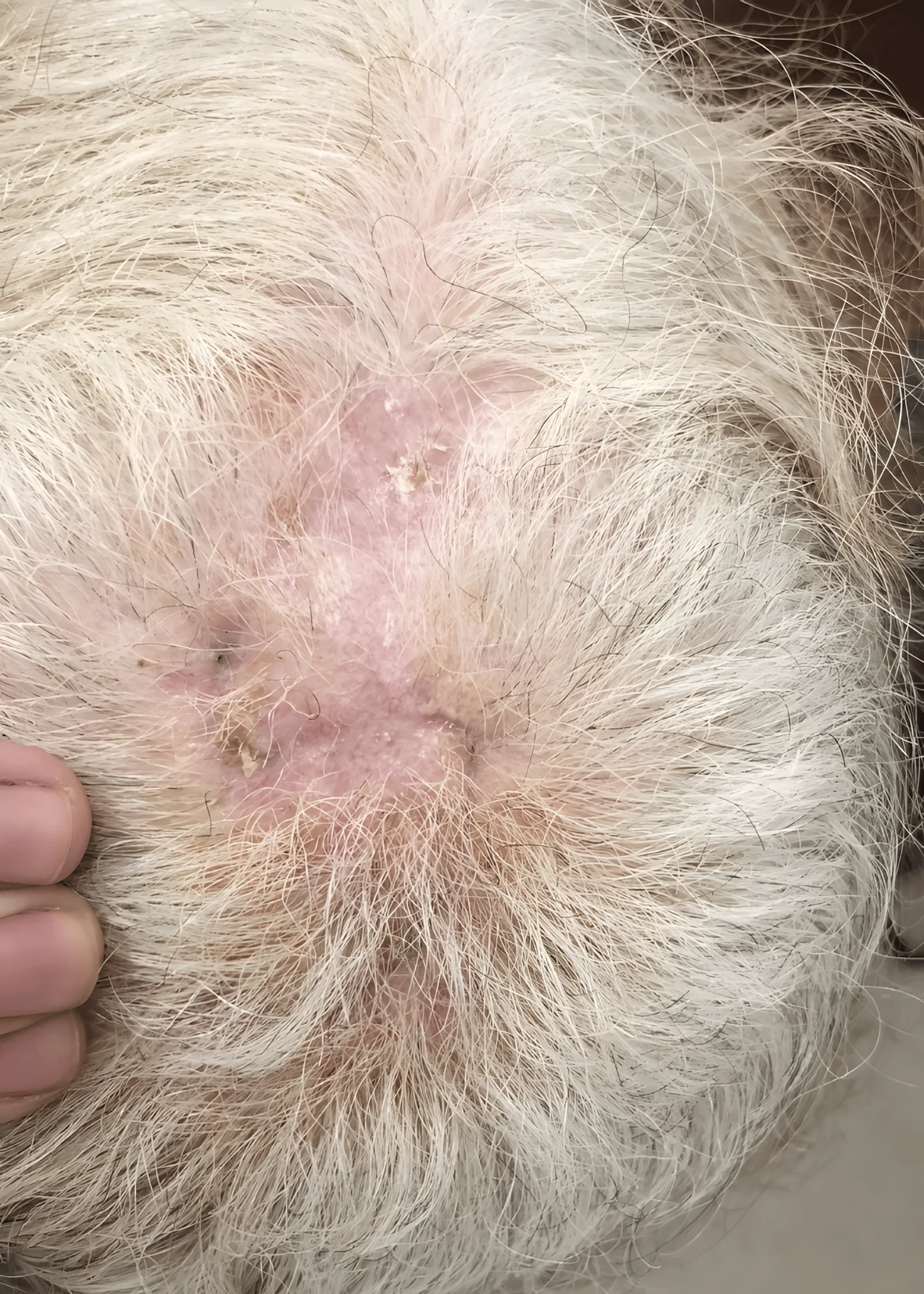
Postoperative day 51 following scalp reconstruction in a 73-year-old patient, demonstrating satisfactory healing without flap necrosis. Mild residual erythema and superficial crusting are present without evidence of active infection or significant wound dehiscence. Hair growth surrounding the reconstruction site provides partial camouflage of the surgical scar.

**Table 1:** Comparative clinical and pathological characteristics of three patients undergoing scalp reconstruction with double rotational flaps. Specimen dimensions and scalp thickness measurements were obtained from the macroscopic examination documented in the official pathology reports and should not be interpreted as postresection defect dimensions. The actual reconstructive defect dimensions were not prospectively recorded. Case 2 developed an approximately 1×5 cm area of superficial distal flap necrosis, which was managed conservatively with collagenase ointment and local wound care, with secondary healing achieved by POD 54 without reoperation. BCC: basal cell carcinoma; SCC: squamous cell carcinoma; POD: postoperative day

Case	Age (years)/sex	Scalp type	Histology	Specimen size	Scalp thickness	Complication	Outcome
1	56/M	Hair bearing	Nodular BCC	3.0×2.7 cm	0.9 cm	None	Complete healing
2	88/M	Bald scalp	SCC	2.9×2.8 cm	0.4 cm	Superficial distal flap necrosis (approximately 1×5 cm)	Secondary healing by POD 54
3	73/F	Hair bearing	SCC	2.9×2.5 cm	0.5 cm	None	Complete healing

## Discussion

Local rotational flaps remain a well-established reconstructive option for small- and moderate-sized scalp defects following oncologic resection because they recruit adjacent tissue with similar color, texture, and hair-bearing characteristics while avoiding the donor-site morbidity associated with free tissue transfer [1,2,5,6]. Published clinical series have reported favorable outcomes with local scalp flaps when appropriate flap design, preservation of vascular supply, adequate tissue mobilization, and minimization of closure tension are achieved [17-20].

As summarized in Table 2, published studies of local scalp flap reconstruction have primarily focused on defect size, flap geometry, reconstructive algorithms, vascular anatomy, and surgical technique [4,7,17-20]. These studies provide important information regarding flap selection and postoperative outcomes; however, advanced androgenetic alopecia and age-related scalp tissue characteristics have not been systematically evaluated as independent variables influencing local flap healing. Therefore, the potential reconstructive relevance of these characteristics remains uncertain.

**Table 2:** Comparison of published clinical series of local scalp flap reconstruction and consideration of scalp tissue characteristics. The table summarizes patient populations, reconstructive techniques, reported postoperative complications, and whether scalp tissue characteristics or androgenetic alopecia were considered in relation to reconstructive outcomes. Previous studies have primarily focused on defect characteristics, flap design, reconstructive techniques, and postoperative complications [4,8,17,19,20]. Although baldness has been discussed in relation to scalp skin cancer risk, its potential association with local flap healing has not been specifically evaluated in these studies [17]. The present three-patient case series is included for comparison; scalp tissue characteristics are considered only as a hypothesis-generating variable, and no causal relationship among androgenetic alopecia, scalp tissue characteristics, and flap viability can be established.

Studies	Patients	Local rotational flaps	Complications	Consideration of scalp tissue quality or androgenetic alopecia
Hoffmann [4]	54	Yes	Low; flap necrosis not specifically reported	No
Mueller et al. [8]	68	Yes	Not specifically reported	No
Russo [17]	119	Yes	10.9% overall complications	Mentioned baldness as a risk factor for scalp skin cancer but did not evaluate its influence on reconstruction
Seretis et al. [19]	138	Yes	9.4% complications; 3.6% reoperations	No
Lopez-Alvarado et al. [20]	18	Yes (7 double rotational flaps)	No flap necrosis or dehiscence	No
Present study	3	Double rotational flaps	One superficial distal flap necrosis treated conservatively	Scalp tissue characteristics considered as a hypothesis-generating variable; no causal association established

Local flap viability depends on multiple interacting patient- and procedure-related factors, including adequate perfusion, preservation of the subdermal vascular plexus, tissue mobility and elasticity, flap design, and closure tension [1-7]. In the present series, the two older patients had relatively similar measured scalp thickness (0.4 cm and 0.5 cm) but experienced different postoperative outcomes. The 88-year-old bald man developed an approximately 1×5 cm area of superficial distal flap necrosis, whereas the 73-year-old woman with hair-bearing scalp healed without flap necrosis. This difference represents a clinical observation rather than evidence that hair-bearing status, androgenetic alopecia, or other qualitative scalp characteristics independently determined flap viability.

Aging is associated with well-described changes in skin biology, including dermal thinning, collagen fragmentation, elastin degeneration, fibroblast senescence, extracellular matrix remodeling, and changes in cutaneous vascular density that may adversely affect tissue elasticity and wound healing [9-13]. Androgenetic alopecia is likewise associated with established biological changes, including progressive follicular miniaturization and perifollicular remodeling [14-16]. These histologic and biological findings should, however, be distinguished from their potential reconstructive implications. A clinically meaningful reduction in rotational flap perfusion or viability attributable to androgenetic alopecia has not been established, and vascular perfusion was not objectively measured in the present series. Accordingly, androgenetic alopecia should not be interpreted as an established independent risk factor for local scalp flap necrosis on the basis of these observations.

Although the 73-year-old woman had a measured scalp thickness comparable to that of the 88-year-old bald patient, their different postoperative outcomes cannot be attributed to hair preservation or tissue characteristics alone. Differences in age, sex, closure tension, chronic sun exposure, tumor characteristics, and other patient- or procedure-related factors may have contributed. Importantly, closure in Case 2 was achieved under clinically assessed moderate tension, which represents a plausible technical contributor to distal flap ischemia and necrosis. Although the same general double rotational flap technique was used in all three patients, with approximate flap dimensions of 3.5×6 cm, flap-limited undermining, and galeal scoring, these similarities do not eliminate the potential influence of other technical or patient-specific variables. The observed difference therefore raises the hypothesis that scalp characteristics beyond measured thickness may be relevant to reconstructive outcomes but does not establish their independent influence on flap viability.

The superficial distal flap necrosis in Case 2 remained limited to an approximately 1×5 cm area and was successfully managed with collagenase ointment and local wound care, with secondary healing by postoperative day 54 and no need for reoperation. Limited superficial ischemic complications following local scalp flap reconstruction may be amenable to conservative management in appropriately selected cases [17,19,20]. The favorable secondary healing observed in this patient should therefore be considered separately from the factors that may have contributed to the initial development of necrosis.

Baldness is well recognized as a risk factor for scalp skin cancer because of increased cumulative ultraviolet exposure [14,17]. Its potential relevance to reconstructive outcomes, however, has received considerably less attention. Current reconstructive approaches primarily consider defect characteristics, scalp laxity, vascular anatomy, flap geometry, and technical factors [1,2,5,6,17-20]. Whether biological or structural scalp characteristics associated with aging, androgenetic alopecia, or chronic actinic exposure provide additional clinically useful information for reconstructive planning remains unknown and requires prospective investigation.

The principal limitation of this study is the small sample size inherent to a three-patient case series, which precludes establishing causal relationships between scalp tissue characteristics and postoperative healing. Although the same general double rotational flap technique was used in all three patients, with approximate flap dimensions of 3.5×6 cm, flap-limited undermining, and galeal scoring, the actual postresection defect dimensions were not prospectively recorded. Closure tension was assessed clinically rather than objectively, and moderate closure tension documented in Case 2 represents a potential technical confounder. In addition, vascular perfusion and scalp biomechanical properties were not objectively assessed. Therefore, the contribution of individual tissue characteristics to flap viability cannot be isolated from other patient- and procedure-related factors.

Histopathology slides and photomicrographs could not be included because pathological processing, slide preparation, and diagnostic evaluation were performed by an external pathology laboratory. The original slides were not available to the author during manuscript preparation. Therefore, the histopathological diagnoses and pathological characteristics reported in this case series were derived exclusively from the official pathology reports and were not independently re-reviewed by the author. The absence of histopathological photomicrographs represents an additional limitation.

Despite these limitations, the comparison of these three cases raises the hypothesis that scalp characteristics beyond measured thickness may warrant further investigation in reconstructive planning. Prospective studies incorporating objective assessments of scalp perfusion, biomechanical properties, tissue characteristics, and relevant patient- and procedure-related variables are needed to evaluate this hypothesis.

## Conclusions

This three-patient case series raises the hypothesis that factors beyond measured scalp thickness may warrant further investigation in local rotational flap reconstruction. Although different healing outcomes were observed among patients with varying scalp characteristics, the small sample size and potential patient- and procedure-related confounders preclude determining the independent contribution of tissue characteristics, androgenetic alopecia, or scalp thickness to flap viability. These observations are hypothesis-generating and support further prospective investigation incorporating objective assessments of scalp tissue characteristics, perfusion, and relevant clinical and technical variables.
